# Carnitine Palmitoyltransferase 1 Increases Lipolysis, UCP1 Protein Expression and Mitochondrial Activity in Brown Adipocytes

**DOI:** 10.1371/journal.pone.0159399

**Published:** 2016-07-20

**Authors:** María Calderon-Dominguez, David Sebastián, Raquel Fucho, Minéia Weber, Joan F. Mir, Ester García-Casarrubios, María Jesús Obregón, Antonio Zorzano, Ángela M. Valverde, Dolors Serra, Laura Herrero

**Affiliations:** 1 Department of Biochemistry and Physiology, Institut de Biomedicina de la Universitat de Barcelona (IBUB), Universitat de Barcelona, E-08028, Barcelona, Spain; 2 Centro de Investigación Biomédica en Red de Fisiopatología de la Obesidad y la Nutrición (CIBEROBN), Instituto de Salud Carlos III, E-28029, Madrid, Spain; 3 Institute for Research in Biomedicine (IRB Barcelona) and Departament de Bioquímica i Biologia Molecular, Facultat de Biologia, Universitat de Barcelona, E-08028, Barcelona, Spain; 4 Centro de Investigación Biomédica en Red de Diabetes y Enfermedades Metabólicas Asociadas (CIBERDEM), Instituto de Salud Carlos III, E-28029, Madrid, Spain; 5 Instituto de Investigaciones Biomédicas Alberto Sols (CSIC-UAM) and Instituto de Investigación Sanitaria La Paz, 28029, Madrid, Spain; University of Santiago de Compostela School of Medicine - CIMUS, SPAIN

## Abstract

The discovery of active brown adipose tissue (BAT) in adult humans and the fact that it is reduced in obese and diabetic patients have put a spotlight on this tissue as a key player in obesity-induced metabolic disorders. BAT regulates energy expenditure through thermogenesis; therefore, harnessing its thermogenic fat-burning power is an attractive therapeutic approach. We aimed to enhance BAT thermogenesis by increasing its fatty acid oxidation (FAO) rate. Thus, we expressed carnitine palmitoyltransferase 1AM (CPT1AM), a permanently active mutant form of CPT1A (the rate-limiting enzyme in FAO), in a rat brown adipocyte (rBA) cell line through adenoviral infection. We found that CPT1AM-expressing rBA have increased FAO, lipolysis, UCP1 protein levels and mitochondrial activity. Additionally, enhanced FAO reduced the palmitate-induced increase in triglyceride content and the expression of obese and inflammatory markers. Thus, CPT1AM-expressing rBA had enhanced fat-burning capacity and improved lipid-induced derangements. This indicates that CPT1AM-mediated increase in brown adipocytes FAO may be a new approach to the treatment of obesity-induced disorders.

## Introduction

Obesity is a major public health problem and a worldwide epidemic contributing to the development of associated pathological conditions, such as insulin resistance, type 2 diabetes, cardiovascular disease, nonalcoholic fatty liver disease, and some forms of cancer among others [1,2]. Adipose tissue has gained a crucial role in the study of the mechanisms involved in obesity-related disorders. Adipose tissue is classified into energy-storing white adipose tissue (WAT) and brown adipose tissue (BAT), which controls thermogenesis by burning fatty acids (FA) to produce heat and defend the body against cold. Traditionally, WAT has been implicated in the pathogenesis of obesity-induced insulin resistance [2–5]. BAT has received less attention, because it is less abundant, and it was considered exclusive to rodents and human neonates. However, in the last decade, active BAT was discovered in adult humans by a combination of positron-emission tomography (PET) and computed tomography (CT) (PET-CT) [6–12]. Furthermore, it was shown that BAT is reduced in obese and diabetic patients [8]. Thus, BAT has become a leading research topic, since any strategy that can enhance BAT mass or activity may be a potential therapeutic approach to obesity-induced diabetes.

The cellular heterogeneity of fat is still under debate [13]. To date, at least two types of thermogenic adipocytes have been described in humans and rodents: the classical brown adipocytes, and beige (also called brite) adipocytes. They differ in both their developmental origin and their anatomical localization [14]. Beige adipocytes are located within the WAT and appear in response to certain stimuli such as cold exposure or β3-adrenergic signals. A recent study has identified *Zic1* and *Tcf21* as specific markers for brown and white adipocytes, respectively, while *Cd137*, *Epsti1*, *Tbx1*, and *Tmem26* would be specific for beige adipocytes [15].

Any strategy that can reduce lipid accumulation would be beneficial for the treatment of obesity and related disorders. Studies in rodents have demonstrated that BAT modulates triglyceridemia and controls lipid clearance [16–18]. The aim of our study was to enhance the thermogenic capacity of BAT by increasing its fatty acid oxidation (FAO). Our group and others have shown that increased FAO in white adipocytes and macrophages [19], liver [20–22], pancreas [23,24], neurons [25] and muscle [26–28] results in an improvement in lipid-induced derangements. Cold stimulates FAO in mice, which indicates that FAO is required for thermogenesis [29].

Here we focused on the FAO key enzyme, carnitine palmitoyltransferase 1 (CPT1). CPT1 facilitates the transfer of FAs into the mitochondria for oxidation. There are three CPT1 isoforms, with differences in kinetics, sensitivity to their physiological inhibitor malonyl-CoA, and tissue expression: CPT1A (liver, intestine, kidney, ovary, pancreas, brain and mouse and human WAT), CPT1B (BAT, heart, skeletal muscle and rat and human WAT) and CPT1C (brain and testis) [30,31]. Interestingly, BAT CPT1 activity is decreased in diabetic rats [32]. Thus, we attempted to overexpress a permanently active mutant form of CPT1A, CPT1AM, which is insensitive to malonyl-CoA [33], in a brown adipocyte rat cell line (rBA) through adenoviral infection. The choice of CPT1AM was based on the following points: 1) the CPT1A isoform has lower sensitivity to malonyl-CoA inhibition and higher affinity for the substrate than CPT1B [34]; 2) CPT1A could be differentiated from the BAT endogenous main isoform, CPT1B, and 3) CPT1AM is a permanently active mutant that would ensure constant high levels of FAO, independently of the levels of malonyl-CoA, which is usually derived from glucose metabolism and is the first intermediate in lipogenesis. This last point is particularly relevant in obesity, where in addition to high lipid levels high glucose (and therefore high malonyl-CoA) levels are present.

Here we demonstrate that CPT1AM-expressing rBA display higher FAO, lipolysis, uncoupling protein 1 (UCP1) expression, and mitochondrial respiration. Enhanced FAO in palmitate-incubated rBA reduced triglyceride content and the expression of fatty acid binding protein 4 (FABP4) and tumor necrosis factor α (TNFa), obese and inflammatory markers, respectively. We conclude that CPT1AM-expressing rBA had enhanced lipolysis and UCP1 protein expression, considered as indicators of increased thermogenesis, and showed an improvement in lipid-induced derangements. This highlights CPT1AM-mediated BAT FAO as a new approach to treat obesity and related disorders.

## Materials and Methods

### Materials

Norepinephrine, sodium palmitate, sodium oleate and FA-free BSA were purchased from Sigma Aldrich (St. Louis, MO). DMEM, FBS and penicillin/streptomycin mixture were purchased from Life Technologies (Carlsbad, CA). Etomoxir was kindly provided by Dr. H. P. O. Wolf (GMBH, Allensbach, Germany).

### Cell culture and differentiation of brown adipocytes

Immortalized rBA from neonatal rats were generated as previously described [35]. rBA preadipocytes were grown in DMEM supplemented with 10% fetal serum, 1% penicillin/streptomycin and 2 mM HEPES. rBA were differentiated in DMEM supplemented with 10% fetal serum, 1 nM T3 and 20 nM insulin (differentiation media) until confluence (day 0). Then, cells were incubated with induction medium containing 1 μM rosiglitazone, 0.5 μM dexamethasone, 0.125 μM indomethacin and 0.5 mM isobutylmethylxanthine (IBMX) for two days. Finally, cells were incubated for a further three days in differentiation media, after which they exhibited a fully differentiated phenotype with numerous multilocular lipid droplets in their cytoplasm.

### Oil Red O staining

rBA were grown and differentiated on coverslips. They were rinsed twice in PBS, fixed in 10% paraformaldehyde for 30 minutes at room temperature, and washed again in PBS. Then, cells were rinsed in 60% isopropanol for 5 min to facilitate the staining of neutral lipids, and stained with filtered Oil Red O working solution (0.3% Oil Red O in isopropanol) for 15 minutes. After several washes in distilled water, the coverslips were removed and fixed with mount medium. The intracellular lipid vesicles stained with Oil Red O were identified by their bright red color under the microscope.

### Adenovirus (Ad) infection

At day 6 of differentiation, rBA were infected with AdGFP (100 moi) and AdCPT1AM (100 moi) for 48 hours in serum-free DMEM, 1% penicillin/streptomycin and 2 mM HEPES. Adenovirus infection efficiency was assessed in AdGFP-infected cells.

### Western blot analysis

rBA were cultured in 12-well plates, differentiated and infected as described above. Cells were harvested in lysis buffer (RIPA), and protein concentration was determined using a BCA protein assay kit (Thermoscientific). Samples were separated on 8% and 12% SDS-PAGE gels, for CPT1A and UCP1 respectively, and then transferred onto PVDF membranes (Millipore). The following primary antibodies were used: CPT1A (1/6,000) [24], Tim 44 (1/5,000; BD Bioscience), UCP1 (1/1,000; Abcam) and b-actin (I-19; 1/4,000; Santa Cruz). Blots were incubated with the appropriate IgG-HRP-conjugated secondary antibody. Protein bands were visualized using the ECL immunoblotting detection system (GE Healthcare) and developed on an ImageQuant LAS4000 mini Fuji luminescence imagining system. For the analysis of protein expression, bands from at least three independent experiments were quantified by densitometry using Image J analysis software.

### Analysis of mRNA expression by quantitative real-time PCR

Total RNA was isolated from rBA using an Illustra MiniRNA Spin kit (GE Healthcare), and cDNA was obtained using a Transcriptor First Strand cDNA Synthesis kit (Roche), following the manufacturer’s instructions. Relative quantification of mRNA was performed using a LightCycler^®^ 480 instrument (Roche) in 10 μL of reaction medium by using 6.5 ng of cDNA, forward and reverse primers at 100 nM each, and a SYBR Green PCR Master Mix Reagent kit (Life Technologies). Primer pairs are shown in Table 1. The mRNA levels were normalized to those of b-actin and expressed as fold change.

**Table 1:** Quantitative real-time PCR oligonucleotides.

Gene Name	Forward	Reverse
b-actin	5'- AAGTCCCTCACCCTCCCAAAG-3'	5'AAGCAATGCTGTCACCTTCCC-3'
Cidea	5'-TGATATCCGCTGCACAAGC-3'	5'-CACCTGGGCAGCATAGGA-3'
Cd137	5'-AGCTGACAGAGACCCTGTGG-3'	5'-CACATGCACAGGACACCAA-3'
Fabp4	5'-GAGGAGACGAGATGGTGACAA-3'	5'-GCTCATGCCCTTTCGTAAAC-3'
Mt-Atp6	5'-TAAGCATAGCCATCCCCCTA-3'	5'-TTAGTTTGTGTCGGAAGCCTAGA-3'
Mt-Co1	5'-TCGGAACCCTCTACCTATTATTTG-3'	5'-CTCGAATTAGAATACTTAAAGCTGTCC-3'
Mt-Co2	5'-TAAGCATAGCCATCCCCCTA-3'	5'-TTAGTTTGTGTCGGAAGCCTAGA-3'
Mt-Cyb	5'-CCCTAGTACTATTCTTCCCAGACCT-3'	5’-AGGGGGTTAGCGGGTGTAT-3'
Mt-Nd1	5'-CCTCAACCTAGGCATACCATTT-3'	5’-AGGCTCATCCCGATCATAGA-3'
Pgc1a	5'- AAAGGGCCAAGCAGAGAGA-3'	5'- GTAAATCACACGGCGCTCTT-3'
Prdm16	5'-CGGATGTTCCCCAACAAAT-3'	5'-ACGCTCTTCTGTGTGGACAA-3'
Tnfa	5'-AAATGGGCTCCCTCTCATCAGTTC-3'	5'-TCTGCTTGGTGGTTTGCTACGAC-3'
Zic1	5'-AACCTCAAGATCCACAAAAGGA-3'	5'-CCTCGAACTCGCACTTGAA-3'
Cpt1a*	5'- ACAATGGGACATTCCAGGAG-3'	5'- AAAGACTGGCGCTGCTCA-3'
Cpt1b	5'- GTGACTGGTGGGAAGAGTACG-3'	5'- CTGCTTGTTGGCTCGTGTT-3'
Cpt1c	5'- GCCTGCCAATTTGTGAGAG-3'	5'- GGCAAGGCACTGTTGGTC-3'
Mfn2	5’-ATTGGCCACACCACCAAT-3’	5’-TGGGCTAGCTGGTTCACG-3’

* Recognizes both CPT1A and CPT1AM

### Fatty acid oxidation assay

The FAO rate was measured in rBA grown in 25-cm^2^ flasks, differentiated and infected as described above. On the day of the assay, cells were washed in KRBH 0.1% BSA (FA free), pre-incubated at 37°C for 30 min in KRBH 1% BSA, and washed again in KRBH 0.1% BSA. Cells were then incubated for 3 hours at 37°C with fresh KRBH containing 11 mM glucose, 0.8 mM carnitine plus 0.2 mM [1-^14^C] oleate (Perkin Elmer). Oxidation was measured as previously described [21].

### Glycerol release

rBA were seeded, differentiated in 12-wells plates and infected as described above or incubated in the presence or absence of 5 μM norepinephrine and 180 μM etomoxir for 6 hours. Then, the cell medium was removed and glycerol levels were measured using the TG Triglyceride kit (Sigma-Aldrich), according to the manufacturer's instructions. Samples were normalized by the protein concentration present in each well.

### Lipid extraction from rBA

rBA were seeded, differentiated in 12-wells plates and infected as described above. Then, cells were collected for lipid extraction following Gesta *et al* protocol [36] with minor modifications: after cell lysis with 0.1% SDS, 1/2/0.12 (v/v/v) methanol/chloroform/0.5M KCl solution was added, the two phases were separated by centrifugation and the upper phase was dried with N_2_. Finally, lipids were resuspended in 100% isopropanol.

### Measurement of nonesterified fatty acids

Nonesterified fatty acids (NEFAs) were measured in rBA lipid extracts by NEFA-HR detection kit (Wako Diagnostics) according to the manufacturer’s instructions. NEFA levels were normalized against protein content.

### Seahorse bioanalyzer

The Seahorse XF24 (Seahorse Bioscience, www.seahorsebio.com) was used to measure oxygen consumption rate (OCR) in mature brown adipocytes. For a typical bioenergetic profile we used oligomycin to block ATP synthase; the uncoupler carbonyl cyanide-4-(trifluoromethoxy)phenylhydrazone (FCCP) to measure maximal respiratory capacity; followed by rotenone, the complex 1 inhibitor, and antimycin-A to leave only non-mitochondrial activity to be measured (all from Sigma-Aldrich). Cells were differentiated in customized Seahorse 24-well plates and infected as described above. Before the measurement, cells were incubated for 1 hour with XF Assay Medium (Seahorse Bioscience) plus 5 mM glucose. During the assay, we injected the following at the final concentrations shown: 0.2 μM oligomycin, 0.2 μM FCCP, 1 μM rotenone, and 1 μM antimycin-A. OCR was calculated by plotting the O_2_ tension of media as a function of time (pmol/min), and data were normalized by the protein concentration measured in each individual well. The results were quantified as the average of 8–10 wells +/- SEM per time point in at least three independent experiments.

### Fatty acid (FA) treatment

Sodium palmitate was conjugated with free BSA in a 5:1 ratio to yield a stock solution of 2.5 mM [27]. Cells were incubated with 0.3 mM or 1 mM of this solution or only BSA (basal control) for 24 hours.

### Triglycerides measurement

Triglycerides (TG) were quantified in rBA lipid extracts using TG Triglyceride kit (Sigma), according to the manufacturer's instructions. Protein concentrations were used to normalize sample values.

### Statistical analysis

Data are expressed as the mean ± SEM. Control and experimental groups were compared using Student's *t*-test (column analysis) or two-way ANOVA (grouped analysis). *P*<0.05 was considered statistically significant. All figures and statistical analyses were generated using GraphPad Prism 6 software.

## Results

### Characterization of rBA differentiation stage

**Fig 1:**
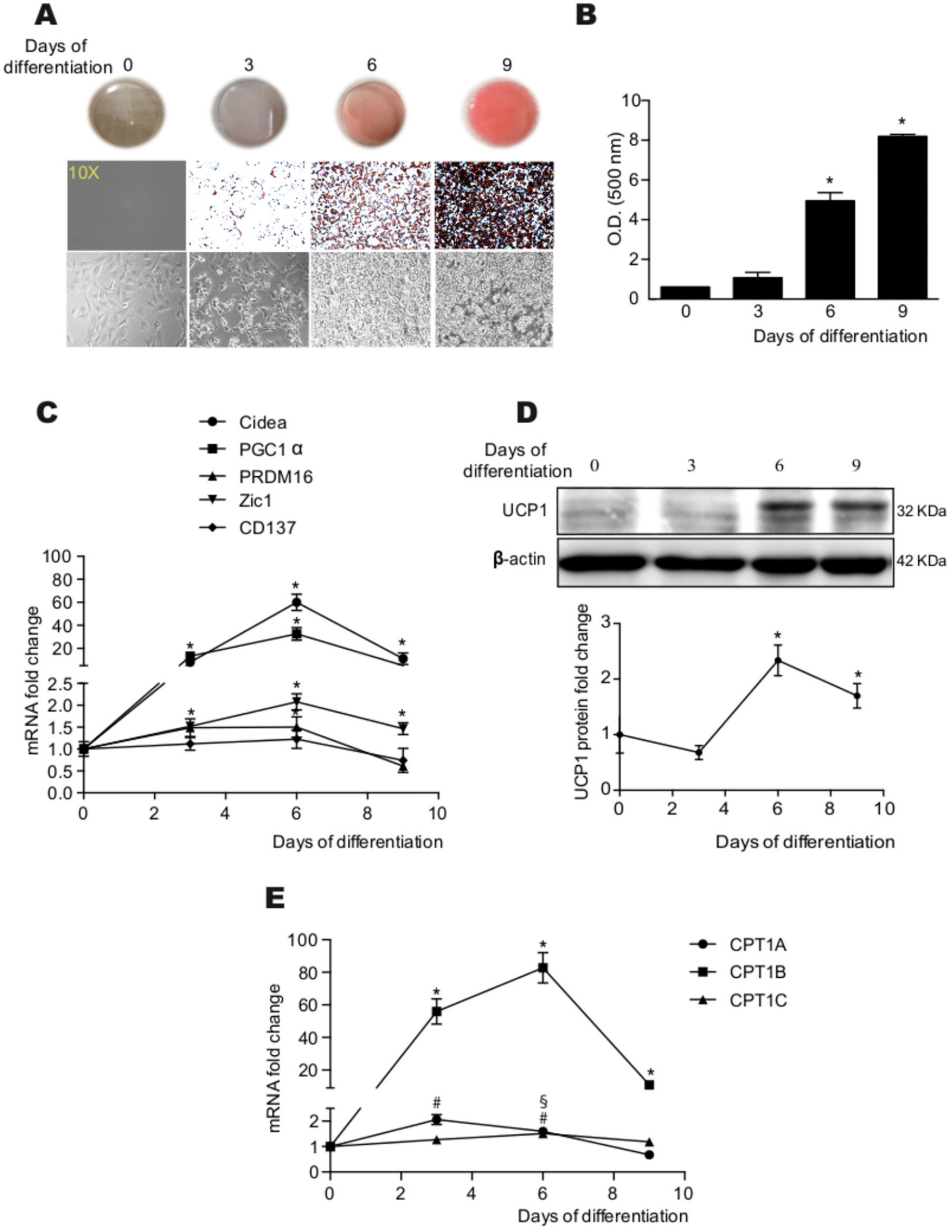
Assessment of rBA differentiation. (A) Oil Red O staining of rBA at different days of differentiation. (B) Quantification of the Oil Red O staining images. (C) Relative mRNA expression of brown (CIDEA, PGC1a, PRDM16, and Zic1) or beige adipocyte markers (CD137). (D) UCP1 protein expression. € Relative mRNA expression of the three CPT1 isoforms during rBA differentiation. The values represent the mean ± SEM of at least three independent experiments. *, #, § *P* < 0.05 compared with day 0 of differentiation of CPT1B, CPT1A and CPT1C, respectively.

A time-course study was performed to assess the differentiation stage of rBA. Differentiation was evaluated by Oil Red O staining, mRNA expression of BAT differentiation markers, and protein levels of UCP1. Oil Red O staining showed an increase in neutral lipid accumulation, starting at day 6 of differentiation (Fig 1A and 1B). The mRNA levels of the brown adipocyte differentiation markers CIDEA, PGC1a, PRDM16 and Zic1 increased at day 3, and peaked at day 6 of differentiation (Fig 1C). The mRNA levels of the beige adipocyte marker CD137 were unchanged (Fig 1C), which indicates that rBA did not show a beige phenotype. The proteins levels of the classical BAT marker, UCP1, were maximal at day 6 of differentiation (Fig 1D). Therefore, day 6, when cells were completely differentiated and had reached maturity, was chosen to perform the expression of CPT1AM.

In addition, we analyzed the mRNA expression pattern of CPT1 isoforms during differentiation (Fig 1E). As expected, CPT1B (the isoform most expressed in BAT), showed higher mRNA changes than the other two isoforms starting at day 3 of differentiation. CPT1A was also increased at day 3 and 6 of differentiation compared to day 0, but to a lesser extent than CPT1B. At day six of differentiation, the three isoforms were significantly increased compared to undifferentiated rBA (day 0).

### Enhanced FAO in CPT1AM-expressing rBA

**Fig 2:**
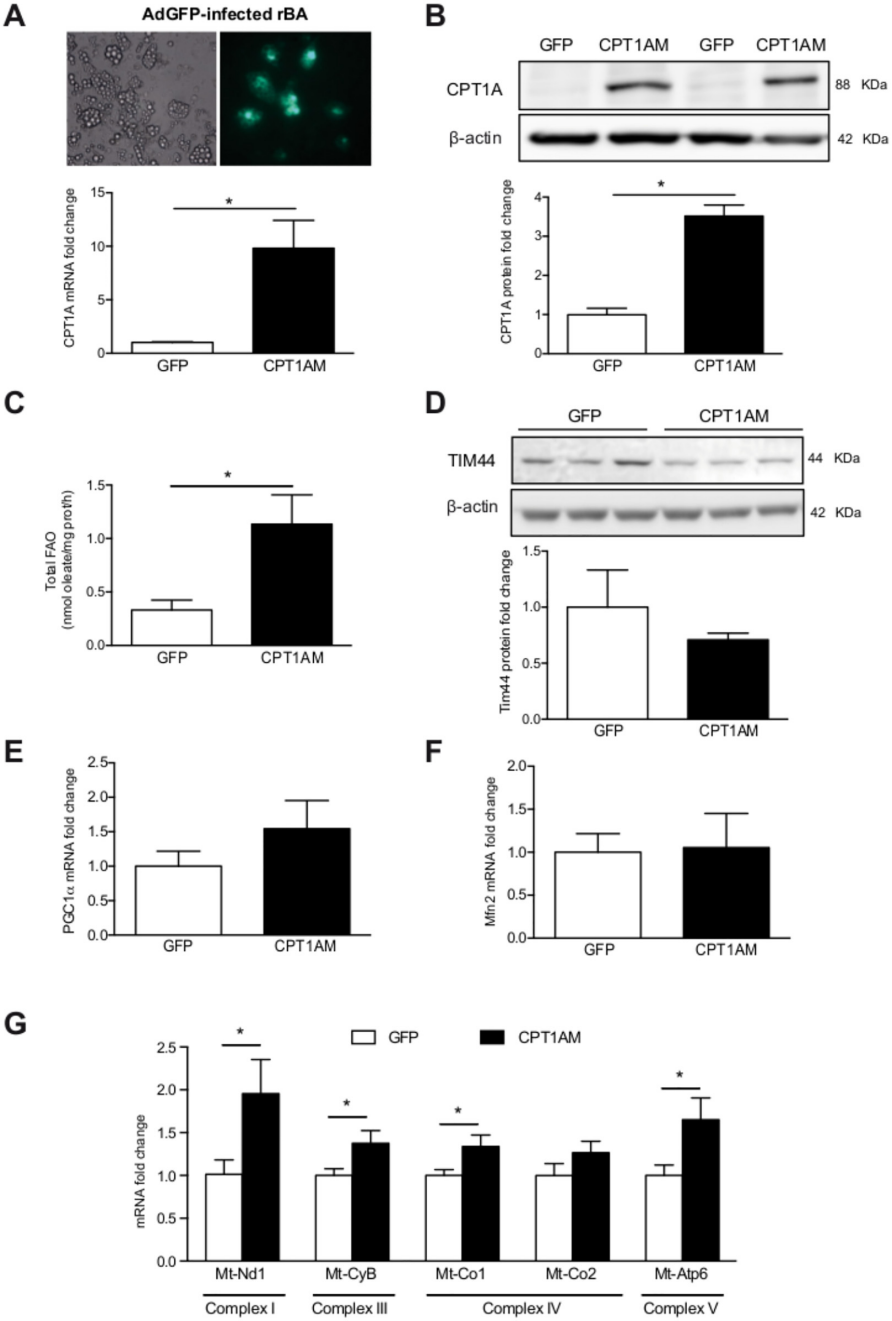
Enhanced FAO in CPT1AM-expressing rBA. (A) (upper panel) AdGFP-infected rBA 48h after infection. A (lower panel): Relative CPT1A mRNA expression in AdGFP- or AdCPT1AM-infected rBA. (B) Whole cell lysates from GFP- and CPT1AM-expressing rBA were subjected to immunoblot analysis with a specific antibody against CPT1A and b-actin. (C) Total FAO rate represented as the sum of acid soluble products plus CO_2_ oxidation. (D) Whole cell lysates from GFP- and CPT1AM-expressing rBA were subjected to immunoblot analysis with a specific antibody against Tim 44 and b-actin. (E-F) Relative PGC1a and Mfn2 mRNA expression in AdGFP- or AdCPT1AM-infected rBA. (G) Relative mRNA expression of the mitochondrial ETC complexes. Shown representative experiment out of 3, n = 3–4. *P < 0.05.

Fully differentiated and mature rBA (day 6) were infected with adenoviruses carrying the CPT1AM gene or GFP as a control. The efficiency of the infection was evaluated in rBA transduced with AdGFP (Fig 2A upper panel; 52.8% infected cells). CPT1A mRNA and protein levels were 9.82- and 3.52-fold higher, respectively, in CPT1AM-expressing rBA than in GFP control cells (Fig 2A lower panel and Fig 2B). The mRNA expression levels of the other CPT1 isoforms (CPT1B and CPT1C) were unaltered in CPT1AM-expressing rBA (S1A Fig and S1 Table). In turn, the FAO rate was 3.42-fold higher in CPT1AM-expressing rBA (Fig 2C). To evaluate whether the increase in FAO was related to an increase in mitochondrial content, we measured the expression of Tim 44 (translocase of mitochondrial inner membrane 44, a mitochondrial content marker), PGC1aα (a mitochondrial biogenesis marker) and Mfn2 (target gene of PGC1a). No changes were seen in any of these markers, which indicate that CPT1AM expression did not affect mitochondrial content or mitochondrial biogenesis (Fig 2D–2F). Interestingly, the mRNA expression of the mitochondrial electron transport respiratory chain (ETC) complexes such as mitochondrial NADH dehydrogenase 1 (Mt-Nd1, complex I), cytochrome b (Mt-Cyb, complex III), cytochrome C oxidase I (Mt-Co1, complex IV component), and ATP synthase 6 (Mt-ATP6, complex V), were increased in CPT1AM-expressing rBA (Fig 2G).

### CPT1AM expression increases lipolysis and UCP1 protein levels

**Fig 3:**
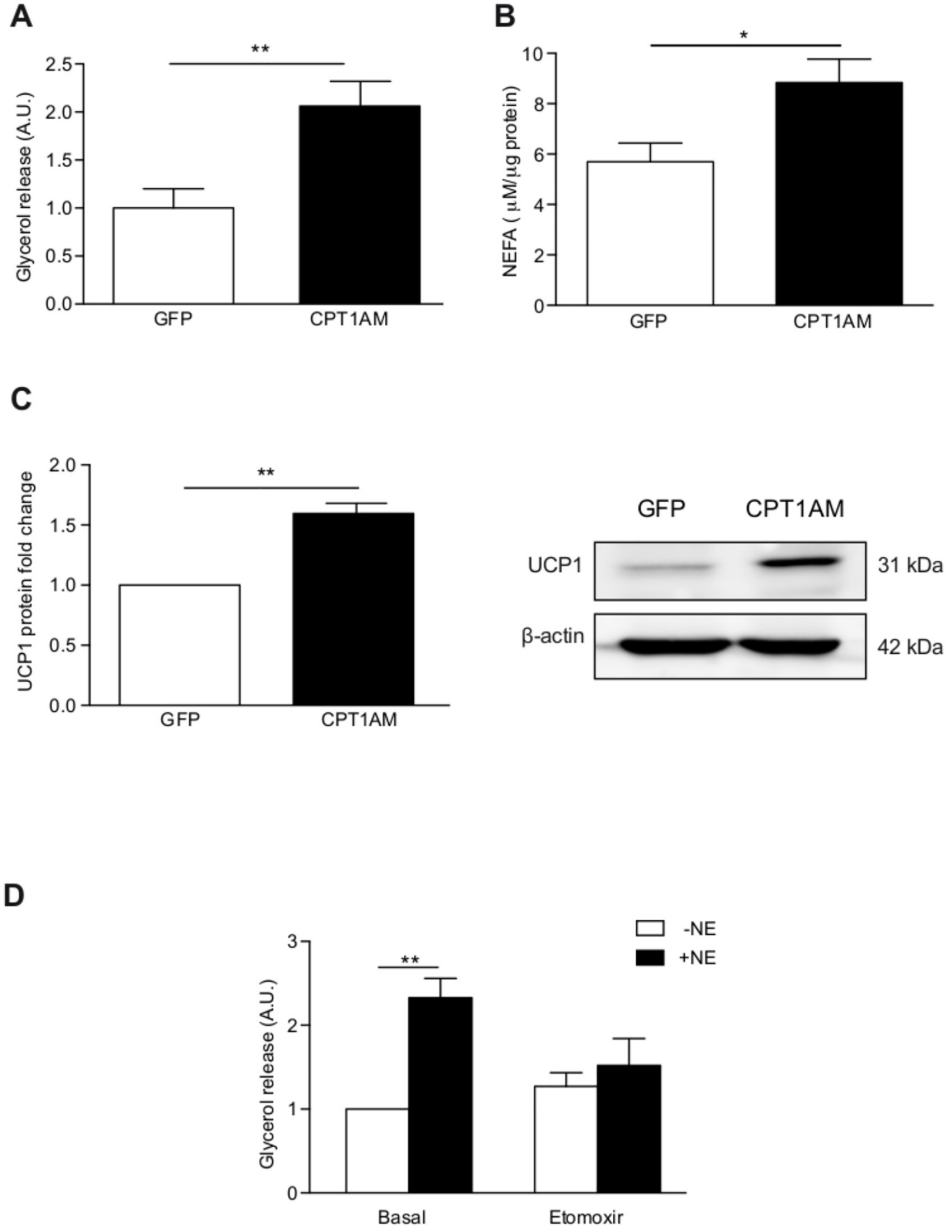
Enhanced lipolysis and UCP1 protein levels in CPT1AM-expressing rBA. (A, B) Lipolysis measured as glycerol release and intracellular NEFAs concentration in GFP- and CPT1AM-expressing rBA. (C) CPT1AM expression increases UCP1 protein levels. (D) Glycerol release in rBA incubated for 6 hours with or without 5 μM norepinephrine (NE) and 180 μM etomoxir. Shown representative experiment out of 3, n = 3–4. *P < 0.05, **P < 0.01.

The effects of increased FAO on lipolysis and UCP1 protein levels were measured as markers of activation of thermogenesis. We measured intracellular nonesterified fatty acids (NEFAs) and glycerol release, which are two major products of lipolysis, a conventional method to quantify thermogenesis. NEFAs and glycerol levels were increased in CPT1AM-expressing rBA, indicating that CPT1AM expression stimulates lipolysis (Fig 3A and 3B). CPT1AM-expressing cells showed a 1.6-fold increase in UCP1 protein levels compared to GFP-infected cells (Fig 3C).

To further examine the role of FAO in rBA thermogenesis, rBA were stimulated with norepinephrine (NE) and incubated with or without etomoxir, an irreversible CPT1-specific inhibitor. Glycerol release was measured after 6 hours of treatment. The NE-induced increase in lipolysis was blunted in etomoxir-treated rBA (Fig 3E).

### Increased mitochondrial respiration in CPT1AM-expressing rBA

**Fig 4:**
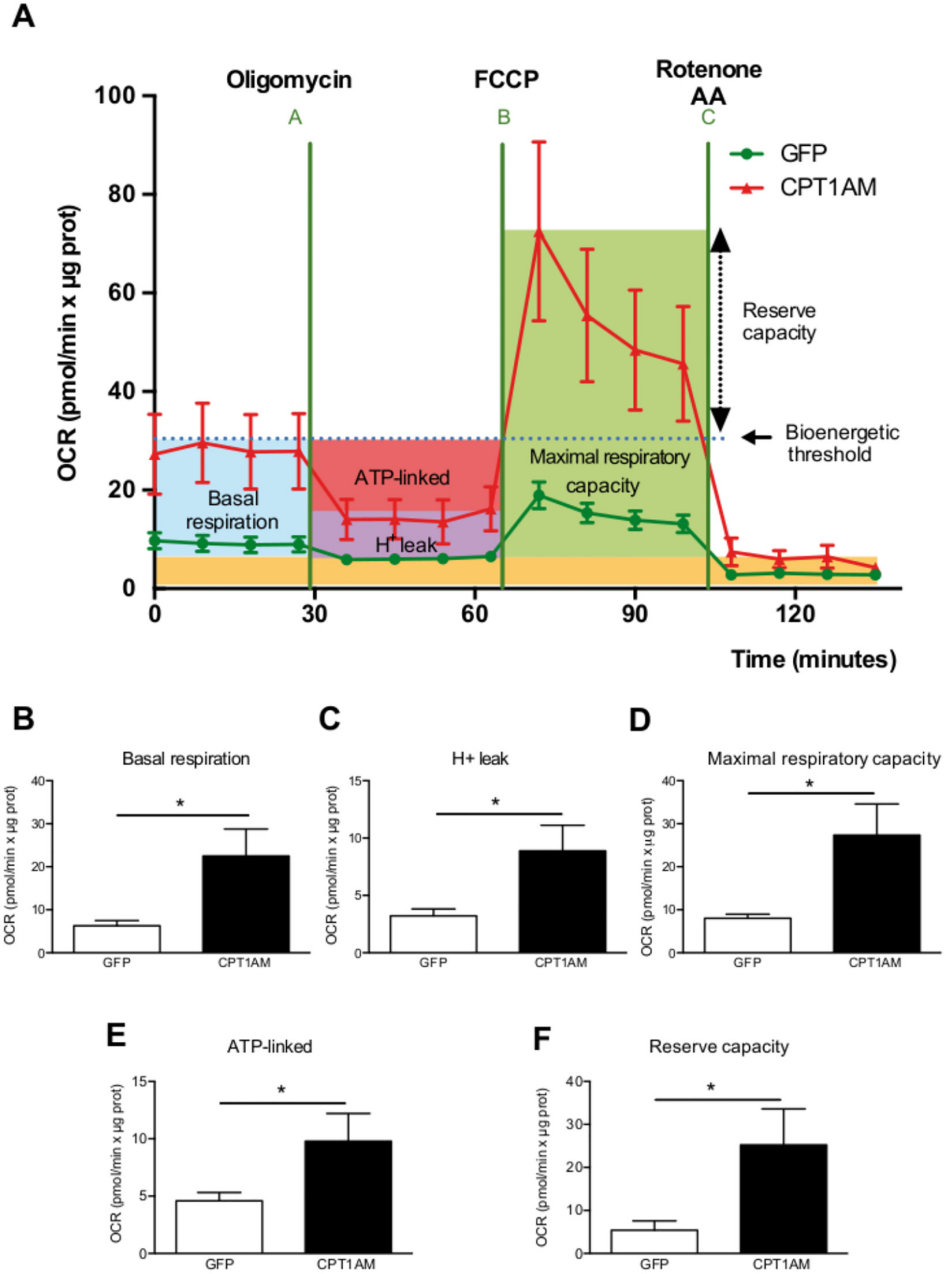
Increased mitochondrial activity in CPT1AM-expressing rBA. (A) Bioenergetic profile of GFP- and CPT1AM-expressing rBA. (B-F) Quantification of Seahorse analysis: basal respiration (B), H^+^ leak (C), maximal respiratory capacity (D), ATP-linked (E) and reserve capacity (F). Shown representative experiment out of 3, n = 8–10. *P < 0.05.

The CPT1AM-induced increase in FAO, UCP1 protein levels and lipolysis prompted us to evaluate potential changes in rBA energetics. Bioenergetic studies were performed using a XF24 Extracellular Flux Analyzer (Seahorse Biosciences) to measure the mitochondrial activity. First, basal respiration was measured, followed by exposure to oligomycin, an inhibitor of ATP synthase, which allowed the measurement of ATP synthesis-coupled respiration and H^+^ leak. Then, the uncoupler carbonyl cyanide 4-(trifluoromethoxy) phenylhydrazone [FCCP] was added to measure the maximal respiratory capacity, followed by the Complex I inhibitor rotenone and complex III inhibitor antimycin A, which left only non-mitochondrial respiration to be measured (Fig 4A). Interestingly, the bioenergetic profile of CPT1AM-expressing rBA revealed significant increases in overall mitochondrial respiration (Fig 4A–4F). CPT1AM-expressing rBA showed higher basal respiration (Fig 4B), H^+^ leak (Fig 4C), maximal respiratory capacity (Fig 4D), ATP synthesis-coupled respiration (Fig 4E), and reserve capacity (Fig 4F) than GFP control cells.

### CPT1AM expression reduces palmitate-induced derangements

**Fig 5:**
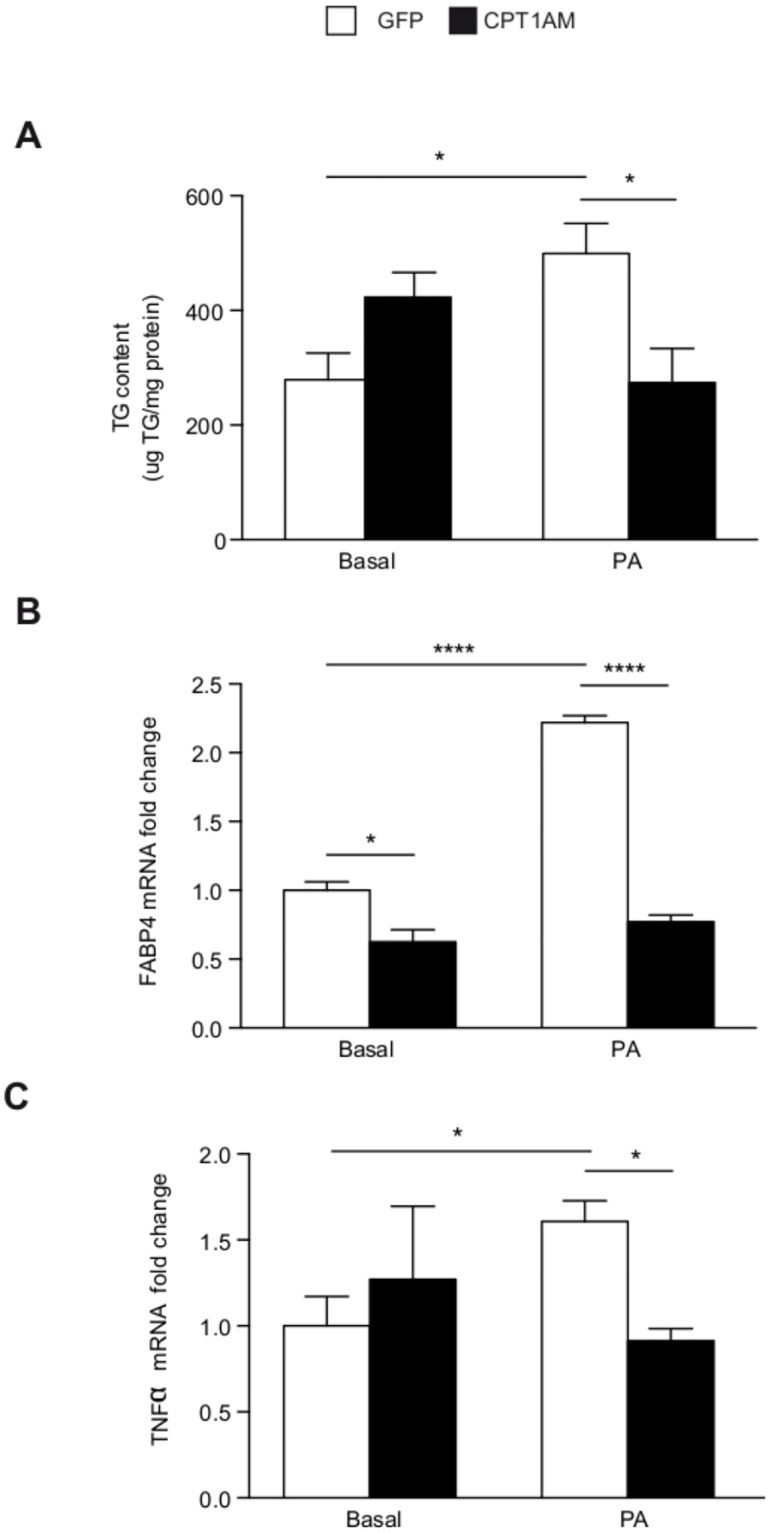
CPT1AM expression is able to reduce lipid-induced derangements. (A) TG content in GFP- or CPT1AM-expressing rBA treated with BSA (basal) or 1 mM palmitate (PA) for 24 hours. (B-C) Relative mRNA expression of FABP4 and TNFa in GFP- or CPT1AM-expressing rBA incubated with 1 mM (FABP4) or 0.3 mM (TNFa) PA. Shown representative experiment out of 3, n = 3–4. *P < 0.05, **P < 0.01.

The palmitate-induced increase in triglyceride (TG) accumulation was blunted in CPT1AM-expressing rBA (Fig 5A). The mRNA levels of the obesity (FABP4) and inflammatory (TNFa) markers were increased after palmitate treatment. However, CPT1AM-expressing rBA were protected from a palmitate-induced increase in FABP4 and TNFa (Fig 5B and 5C). The mRNA levels of the ER stress markers CHOP and EDEM were decreased in CPT1AM-expressing rBA compared to GFP-control cells (S1B Fig and S1 Table).

## Discussion

Obesity and associated diseases such as diabetes have become a worldwide health threat. Dyslipidemia is a common characteristic shared by these metabolic disorders, and the lipid-central point of view has become popular in recent years. The finding that humans have active brown fat has raised high expectations for the treatment of obesity-induced diseases, since brown fat can dissipate caloric energy and reduce both obesity and diabetes in experimental animals [8–12,37–40]. In fact, after short-term cold exposure, brown fat is the main lipid clearance organ [16]. Thus, recent years have seen increasing interest in the regulation of BAT thermogenesis. Most of the strategies have been focused on the role of UCP1 and attempts to enhance heat production, although other authors argue that additional genes may cooperate in the thermogenic function [41].

The finding that BAT is the tissue with the highest FAO rate [42] and that the activity of the FAO rate-limiting enzyme, CPT1, is decreased in BAT of diabetic rats [32] led us to hypothesize that BAT thermogenic power could be enhanced by increasing its FAO. Thus, we overexpressed the constitutively active mutant form, CPT1AM, in rBA.

The assessment of rBA differentiation by Oil Red O staining, UCP1 protein levels and mRNA expression of several BAT differentiation markers showed that the rBA were fully differentiated and mature at day 6. Thus, day 6 was chosen for further experiments. The expression pattern of the three CPT1 isoforms (CPT1A, CPT1B and CPT1C) indicated that all were significantly increased during differentiation, especially CPT1B. Although further studies will be necessary to identify the specific function of each isoform in BAT, these results implicate all three isoforms in the differentiation of brown adipocytes.

Despite BAT being the tissue with the highest FAO rate [42], here we were able to further increase FAO in rBA by the expression of CPT1AM. This was achieved without changes in mitochondrial content or biogenesis. CPT1AM-expressing cells had higher lipolysis and UCP1 protein expression, usually considered as increased thermogenesis. This indicates that CPT1AM-mediated increase in FAO could be an alternative pathway for the regulation of thermogenesis. Initially, BAT thermogenesis requires a plentiful supply of FAs as substrates [43,44]. Therefore, lipolysis is activated during this process, releasing FAs from TG. FAs could be transported into the mitochondria, be involved in the regulation of UCP1 or directly activate UCP1, which uncouples oxidative phosphorylation from ATP production, generating heat [45,46]. Although further experiments will be needed to determine UCP1 regulation during CPT1AM expression, here we have shown that FAO enhancement not only increases lipolysis in rBA, but it is also associated with increased UCP1 protein expression, whose levels are rate-limiting for thermogenesis [47]. Since FA constitute the primary substrate for BAT [48] we hypothesize that CPT1AM expression increases the provision of BAT FA by: 1) directly introducing them into the mitochondria and 2) enhancing lipolysis. The liberated FA then activate and bind to UCP1 in the inner mitochondrial membrane resulting in proton transport, heat generation, and oxygen consumption [48,49]. Thus, CPT1AM may directly or indirectly trigger lipid mobilization to provide BAT mitochondria with fuel (FA).

Several studies have demonstrated the importance of FAO in BAT. Ji *et al*. showed that CPT1B^+/-^ mice developed fatal hypothermia as a result of their inability to perform thermogenesis [50]. More recently, a study in adipocyte-specific CPT2 KO mice indicated that FAO is required for the induction of thermogenic genes in BAT and cold adaptation [29]. In addition, Chondronikola *et al*. have shown that humans with higher amounts of BAT have also higher FAO during cold exposure and that BAT volume is associated with increased lipid metabolism (whole-body FA turnover and oxidation) and adipose tissue insulin sensitivity [51,52]. Here we show that etomoxir (a CPT1-specific inhibitor) blocks TG breakdown in NE-stimulated rBA. These observations indicate that lipolysis supplies substrates for thermogenesis, but when CPT1 is inhibited, free FAs are not transported into the mitochondria and lipolysis is blunted. This would support previous observations that CPT1 inhibitors suppress mitochondrial respiration [53], and that FAO plays an important role during thermogenesis. CPT1AM-expressing rBA displayed increased basal respiration, H^+^ leak, maximal respiratory capacity, and reserve capacity. The increase in H^+^ leak is consistent with the increase seen in UCP1 protein expression. CPT1AM-expressing rBA showed increased lipolysis (FA substrate availability) and mitochondrial ETC complexes mRNA expression. This could have contributed to the enhanced maximal respiratory capacity [54]. Reserve capacity is the difference between maximal respiratory capacity and basal respiration, and it is a useful qualitative indicator of mitochondrial energetic status [54,55]. Thus, our data indicate that enhanced FAO in brown adipocytes potentiates mitochondrial activity.

Finally, we evaluated whether enhanced FAO by CPT1AM expression in the context of obesity would improve lipid-induced derangements. Thus, we decided to mimic an obese phenotype in our cellular rBA model. Traditionally, FA incubation has been used to activate thermogenesis [56]. We tested several palmitate concentrations and different times of incubation to achieve a lipid-induced increase in TG accumulation and FABP4 and TNFa mRNA expression, similar to that previously observed in obese and lipodystrophic mice [16,57,58]. Enhanced FAO in rBA reduced TG content and FABP4 and TNFa mRNA levels, thereby protecting brown adipocytes from obesity and inflammation. These observations highlight CPT1AM-mediated activation of FAO in brown adipocytes as a potential strategy to treat obesity-induced diseases.

In summary, we have shown that CPT1AM expression in rBA increases FAO, lipolysis, UCP1 protein levels and mitochondrial activity. Incubation of rBA with the CPT1-specific inhibitor, etomoxir, blocks NE-stimulated lipolysis. Furthermore, enhanced FAO restored the palmitate-induced increase in TG accumulation and the expression levels of obese and inflammatory markers. We conclude that enhancing the fat-burning power of brown adipocytes through CPT1AM expression may protect them from lipid-induced derangements. Thus, CPT1AM-mediated increase in lipolysis, UCP1 protein expression and mitochondrial activity in brown adipocytes may lead to a new treatment of obesity and related disorders.

## Supporting Information

S1 Fig mRNA expression levels of CPT1 isoforms and ER stress markers. (A) Relative mRNA expression of CPT1A, CPT1B and CPT1C in GFP- or CPT1AM-expressing rBA. (B) Relative mRNA expression of BiP, CHOP and EDEM in GFP- or CPT1AM-expressing rBA incubated with 1 mM palmitate (PA). See S1 Table for primer design. Shown representative experiment out of 3, n = 3–4. *P < 0.05, **P < 0.01.
(TIF)

S1 Table Quantitative real-time PCR oligonucleotides. (DOCX)

## Abbreviations

BAT: brown adipose tissue
CIDEA: cell death-inducing DNA fragmentation factor-α-like effector A
rBA: rat brown adipocyte cells
CHOP: C/EBP homologous protein
CPT1A: carnitine palmitoyltransferase 1A
CPT1AM: permanently active mutant form of CPT1A
EDEM: ER degradation enhancing α-mannosidase-like protein
ER: endoplasmic reticulum
FA: fatty acids
FAO: fatty acid oxidation
Mfn2: mitofusin 2
Mt-ATP6: Mitochondrial ATP synthase 6
Mt-Co1: cytochrome C oxidase I
Mt-Co2: cytochrome C oxidase II
Mt-Nd1: NADH dehydrogenase 1
Mt-Cyb: cytochrome b
NEFA: nonesterified fatty acids
OCR: oxygen consumption rate
PGC1a: peroxisome proliferator-activated receptor gamma coactivator 1 alpha
PRDM16: PR domain-containing 16
TG: triglyceride
Tim 44: translocase of mitochondrial inner membrane 44
UCP1: uncoupling protein 1
WAT: white adipose tissue
