# Supplementary figures and images for: Carnitine Palmitoyltransferase 1 Increases Lipolysis, UCP1 Protein Expression and Mitochondrial Activity in Brown Adipocytes
Source: PLoS One. 2016 Jul 20;11(7):e0159399. doi: 10.1371/journal.pone.0159399 (PMC4954705; doi:10.1371/journal.pone.0159399)

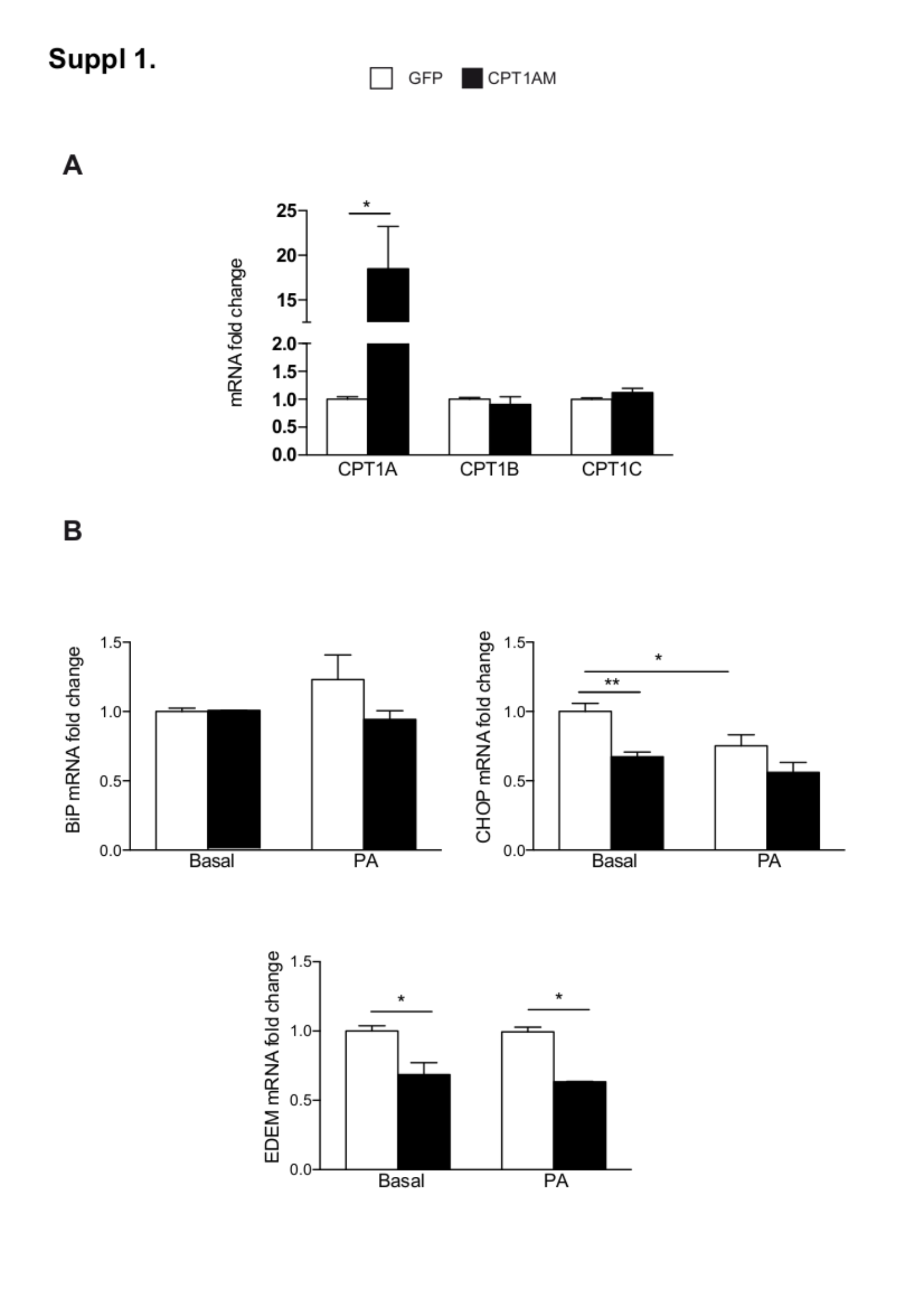

Supplement: S1 Fig — (A) Relative mRNA expression of CPT1A, CPT1B and CPT1C in GFP- or CPT1AM-expressing rBA. (B) Relative mRNA expression of BiP, CHOP and EDEM in GFP- or CPT1AM-expressing rBA incubated with 1 mM palmitate (PA). See S1 Table for primer design. Shown representative experiment out of 3, n = 3–4. *P < 0.05, **P < 0.01. (TIF) [file pone.0159399.s001.tif]
