# Supplementary material for: Carnitine Palmitoyltransferase 1 Increases Lipolysis, UCP1 Protein Expression and Mitochondrial Activity in Brown Adipocytes
Source: PLoS One. 2016 Jul 20;11(7):e0159399. doi: 10.1371/journal.pone.0159399 (PMC4954705; doi:10.1371/journal.pone.0159399)
Supplement: S1 Table — (DOCX) [file pone.0159399.s002.docx]

**S1 Table. Quantitative real-time PCR oligonucleotides**

| **Gene Name** | **Forward** | **Reverse** |
| --- | --- | --- |
| BiP | 5'- CCGTAACAATCAAGGTCTACGA-3' | 5'-AAGGTGACTTCAATCTGGGGTA-3' |
| Chop | 5'- CCAGCAGAGGTCACAAGCAC-3' | 5'- CGCACTGACCACTCTGTTTC-3' |
| Edem | 5'- TGGAATTTGGGATTCTGAGC-3' | 5'- TCTGGATGTTCACAACATTGC-3' |
